# Cancer-induced changes in systemic intravascular monocyte dynamics revealed by MRI single cell tracking in a murine breast cancer model

**DOI:** 10.1186/s41747-026-00831-x

**Published:** 2026-11-03

**Authors:** Asli Havlas, Ina Rebecca Fredrich, Enrica Wilken, Lydia Wachsmuth, Michael Kuhlmann, Christiane Geyer, Emily Hoffmann, Ricarda Jürgens, Thomas Vogl, Cornelius Faber, Anne Helfen, Max Masthoff

**Affiliations:** 1 Translational Research Imaging Center, Clinic for Radiology, University and University Hospital of Muenster, Muenster, Germany; 2 European Institute for Molecular Imaging (EIMI), University of Muenster, Muenster, Germany; 3 Institute of Immunology, University of Muenster, Muenster, Germany

**Keywords:** Cell tracking, Magnetic iron oxide nanoparticles, Magnetic resonance imaging, Neoplasms, Time-lapse imaging

## Abstract

**Objectives:**

In cancer, monocytes with patrolling-like behavior have been associated with antitumoral effects, but their intravascular motion is currently concealed to noninvasive imaging techniques. This study aimed to evaluate systemic monocyte intravascular patrolling-like behavior during tumor growth, immunotherapy, and splenectomy, using single-cell tracking with preclinical time-lapse brain magnetic resonance imaging (MRI).

**Materials and methods:**

In a murine syngeneic breast cancer model (4T1, 67NR), time-lapse MRI (repetitive T2*-weighted gradient-echo sequence after *in vivo* labeling with iron oxide nanoparticles for monocyte detection) of the brain was conducted using a 9.4 T small animal MRI. Control mice were either naive or received sham injections. Additionally, time-lapse MRI was conducted during immunotherapy or following splenectomy in tumor-bearing and tumor-free mice. Individual cells were identified as hypointensities. Tumors were harvested for *ex vivo* analysis.

**Results:**

The number of detected cells within the brain, distant from the tumor, changed during cancer growth. The cell count was significantly lower for tumor-bearing mice compared to controls. In mice bearing 4T1 tumors, the cell count was significantly lower on day 1 and 9 compared to mice bearing 67NR tumors. During immunotherapy, the monocyte count was significantly lower compared to control, but only in the presence of a tumor. Splenectomy reduced the tumor-associated effect on monocyte dynamics in both low- and highly malignant tumors.

**Conclusion:**

Based on an exemplary murine breast cancer model, both cancer and immunotherapy change systemic intravascular monocyte patrolling-like behavior early during tumor growth distant from the tumor, an observation that is dependent on the grade of malignancy and the presence of splenic reservoirs.

**Key Points:**

***Question*** Do tumors alter systemic intravascular monocyte dynamics during tumor growth, immunotherapy, and splenectomy at distant sites, and can such changes be detected noninvasively?

***Findings*** In murine breast cancer, time-lapse MRI revealed reduced monocyte patrolling-like behavior during tumor growth and immunotherapy, influenced by malignancy grade and splenic reservoirs.

***Critical Relevance*** Noninvasive single-cell MRI tracking provides novel insights into systemic immune dynamics in cancer, potentially enabling future monitoring of tumor-immune interactions, therapeutic responses, and immune modulation beyond conventional assessment of tumor burden or tumor microenvironment composition.

**Graphical Abstract:**

**Figure:**
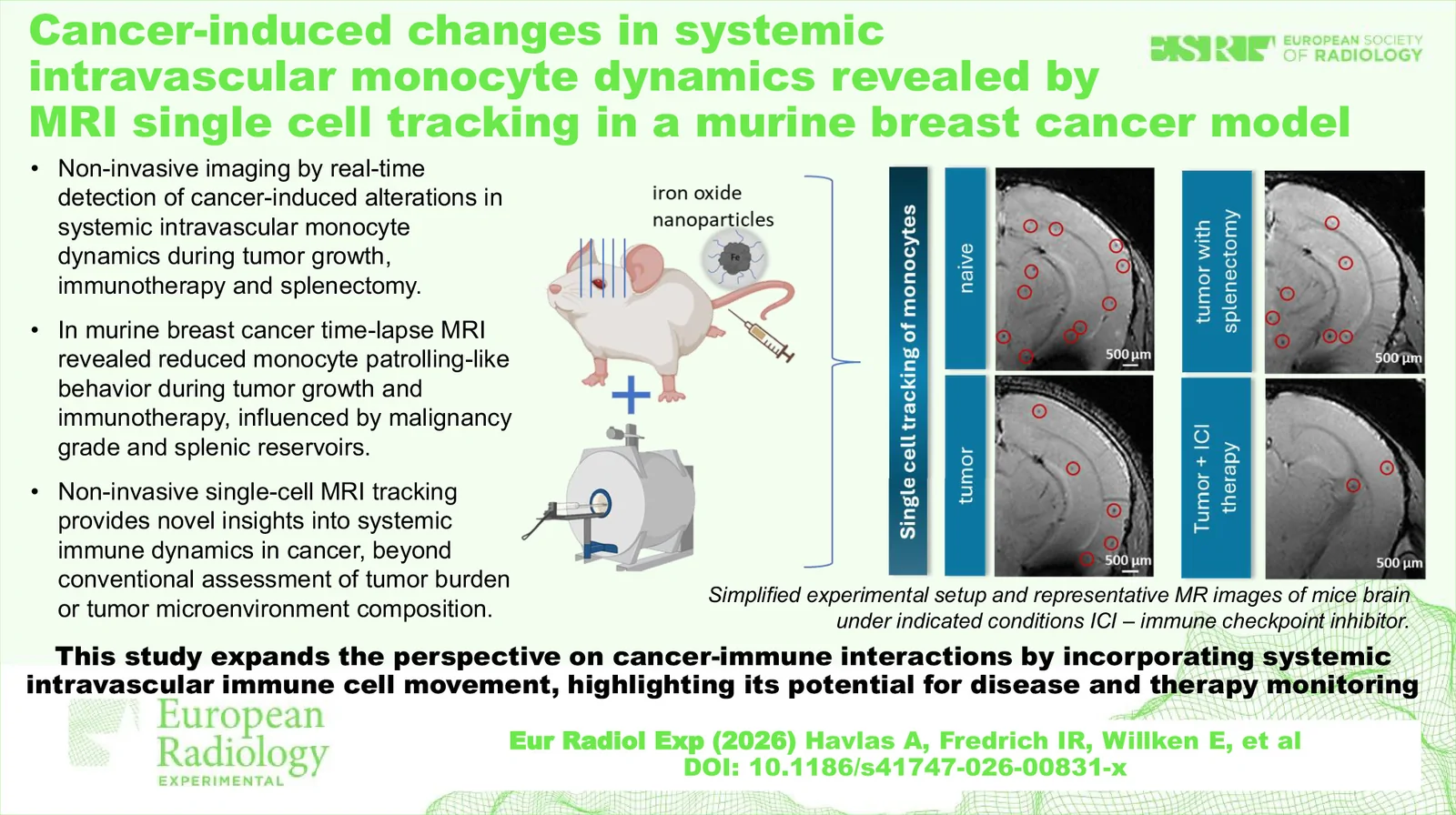

## Introduction

Over the past decades, the understanding of cancer has evolved, viewing the disease as a complex ecosystem of diverse cellular and non-cellular components that interact across multiple scales, shaping the tumor microenvironment (TME) [1, 2]. The active recruitment of immune cells leads to tumor-associated inflammation, which Hanahan and Weinberg have identified as a hallmark of cancer and a critical factor in tumor growth and progression [3]. Consequently, clinical oncology has recognized tumor-associated immune cells as targets for cancer therapy, spurring the development of several cell-specific therapeutic strategies [4, 5]. Among the immune cells within the TME, macrophages, T cells, and neutrophils are prevalent [6]. Tumor-associated macrophages (TAMs) are particularly influential, promoting neoangiogenesis, supporting local invasion through extracellular matrix degradation, and suppressing antitumor immunity [7–9].

TAMs primarily originate from classical “inflammatory” monocytes (CCR2high Ly6C+ in mice, CCR2high CD14 + CD16- in humans), which facilitate early tumor growth and metastasis [10, 11]. Conversely, nonclassical “patrolling” monocytes (pMO; CX3CR1high Ly6Clow/- in mice, CX3CR1high CD14dim CD16+ in humans) are less understood. pMOs are characterized by intravascular motion behavior, slowly patrolling the endothelium in healthy individuals or helping to resolve inflammation in case of a stimulus [12–14]. In cancer, pMOs scavenge tumor material and recruit natural killer cells, reducing the risk of distant metastases [15]. Additionally, higher pMO levels within the TME have been linked to reduced tumor burden in breast cancer models [16]. Thus, pMO emerged as a promising target for cancer diagnosis and treatment (monitoring). We hypothesize that cancer systemically alters the intravascular slow motion behavior of monocytes at distant sites.

To prove this hypothesis, imaging techniques capable of not only assessing static snapshots but also dynamic single-cell motion are needed. Magnetic resonance imaging (MRI) currently best provides a holistic, noninvasive picture of cancer [17]. Specifically, time-lapse MRI using repetitive T2*-weighted imaging, enables dynamic tracking of single iron-labeled monocytes moving at slow velocities consistent with patrolling behavior (here termed “patrolling-like”)—but it does not capture faster-moving cells such as those freely circulating or rolling [18–20]. This motion-based imaging readout provides an approximation of patrolling-like behavior, serving as a surrogate rather than a direct identification of phenotypically defined monocyte subsets. Thus, this technique provides ideal prerequisites to noninvasively reveal the currently concealed patrolling-like intravascular motion of monocytes.

In this study, we used the time-lapse MRI motion-based imaging readout using a murine breast cancer model to study if and how cancer affects intravascular monocyte motion distant from the primary site, in particular with respect to the underlying grade of malignancy, the impact of immunotherapy, or the modulation of the organism’s monocyte reservoirs.

## Materials and methods

### Study design

**Fig. 1:**
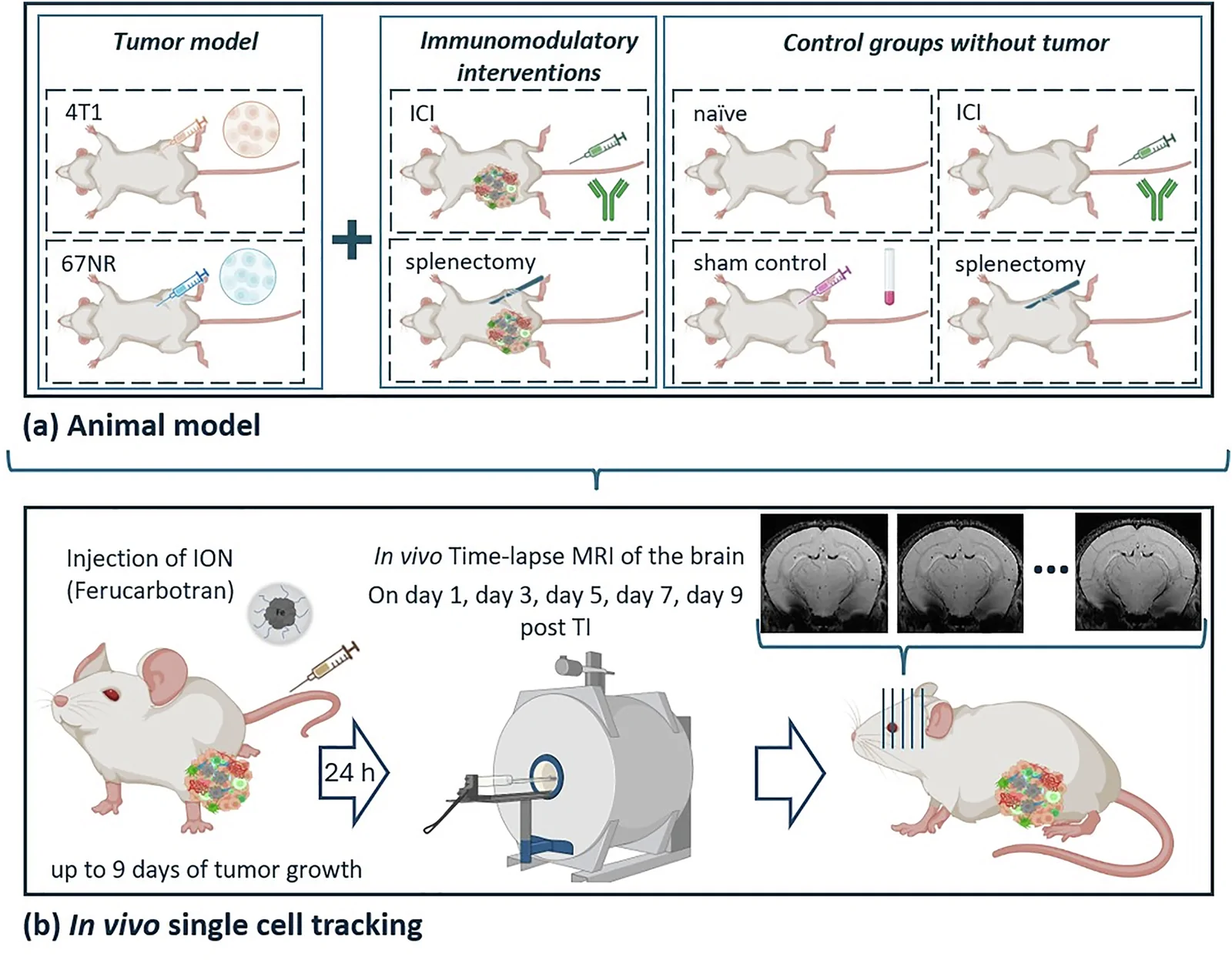
Experimental setup to study intravascular monocyte patrolling-like behavior in cancer. **a** Breast cancer model with either 4T1 (highly malignant) or 67NR (low malignant) cells injected into the mammary fat pad of BALB/c mice. In case of immunomodulatory intervention, either ICI therapy or splenectomy was performed. Control groups included naive mice, sham injection of culture medium into the mammary fat pad, and ICI injection or splenectomy in mice without tumors, respectively. **b** Time-lapse MRI was performed on a 9.4-T small animal magnet with a cryogenic probe 24 h after *in vivo* labeling of monocytes with iron-oxide nanoparticles (Ferucarbotran) between day 1 and 9 after tumor initiation in the aforementioned groups. ICI, Immune checkpoint inhibitor; ION, Iron oxide nanoparticles; TI, Tumor implantation. Partially created using BioRender.com

We followed the stepwise approach outlined in Fig. 1, using time-lapse MRI to detect and follow single monocytes within the brain vasculature in all tested conditions (supplementary Video S1 and baseline scan in supplementary Video S2). First, we studied intravascular monocyte dynamics in the brain (as a surrogate for a tumor-distant vascular bed) during tumor growth in two syngeneic mouse models of breast cancer derived from the same maternal cell line (4T1, high malignant; 67NR, low malignant). Time-lapse MRI was performed on days 1, 3, 5, 7, and 9 after tumor implantation (TI) (*n* = 5 per time point for each tumor cell line). Naive mice without TI served as controls (naive, *n* = 7).

**Fig. 2:**
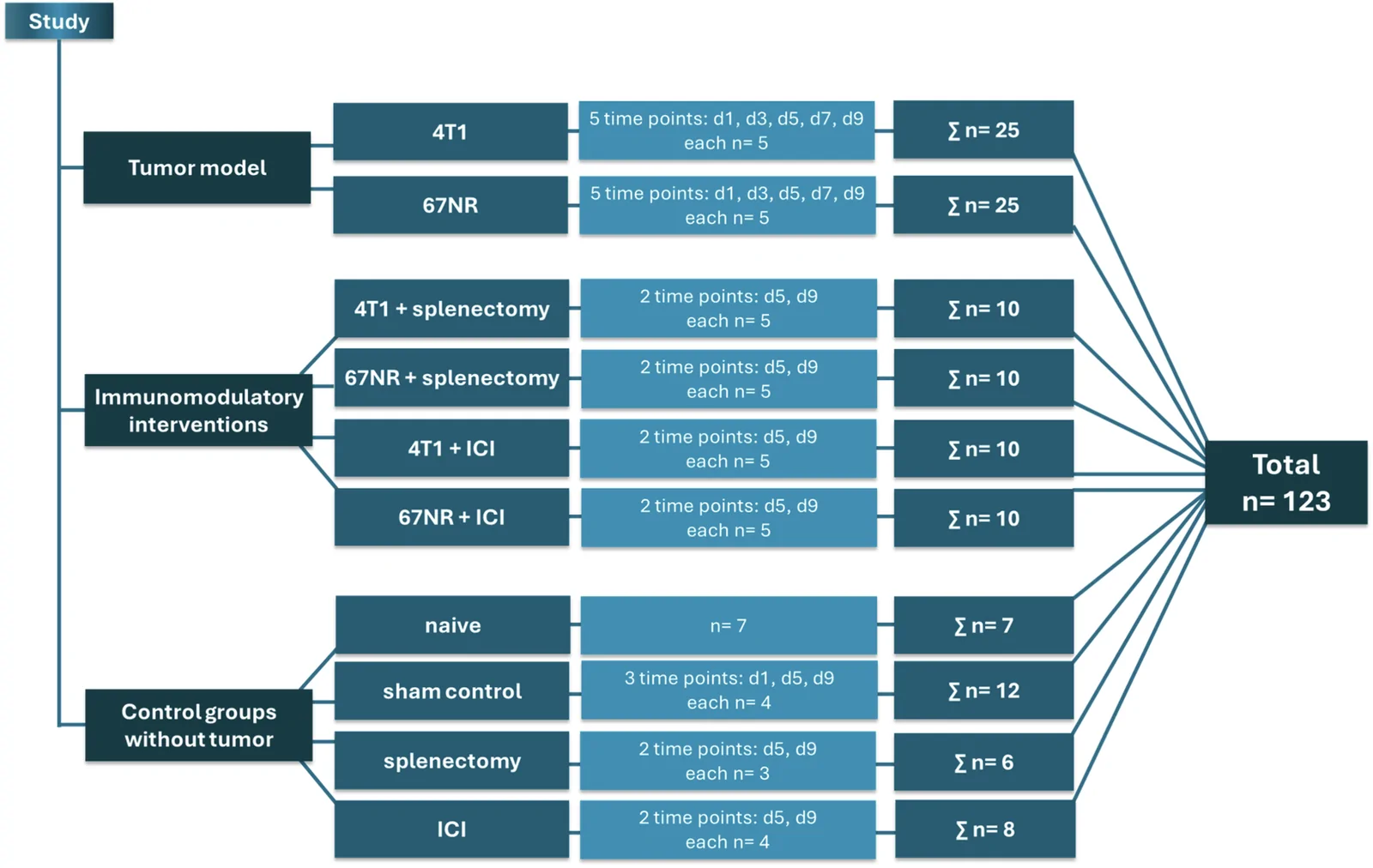
Flowchart of included animals in the study. Overview of animal numbers in each experimental group and the resulting total animal number. Tumor models with graded malignancy (4T1 and 67NR) were further divided according to the immunomodulatory intervention with ICI injections or splenectomy, as well as control groups without tumor, including naive mice, sham control, ICI injection, or splenectomy. All numeric data in the flow charts correspond to the number of mice, which equals the number of MRI readings. ICI, Immune checkpoint inhibitor

Additionally, sham controls received an injection of culture medium without tumor cells into the mammary fat pad (time-lapse MRI on day 1, 5, and 9; *n* = 4 each). Next, we tested whether modulation of the immune system influences intravascular monocyte dynamics during tumor growth. Splenectomy (immediately before TI, day 5 and day 9; *n* = 5 each) was performed, or therapy with immune checkpoint inhibitors (ICI), intravenous injection of anti-PDL1 on day 5 and day 9 (*n* = 5 each), was administered in tumor-bearing mice (4T1, 67NR), with time-lapse MRI performed on day 5 and day 9 after TI. Controls were performed receiving splenectomy (*n* = 3 each) or only ICI therapy without TI (*n* = 4 each), respectively. An overview of the total number of animals and the number of animals in each experimental group is provided as a flow chart in Fig. 2. After MRI, tumors were harvested, and TME composition was analyzed using flow cytometry.

### Animals

Animal husbandry and experiments complied with local guidelines on animal care and were approved by local authorities (ID:81-02.04.2020.A194). Female BALB/c mice (body weight: 18–20 g; age: 8–12 weeks) were examined in this study (Charles River Laboratories, Sulzfeld, Germany) and housed under a 12-h light-dark cycle. They were fed a standard chow diet and provided with food and water ad libitum. Mice were monitored daily for disease symptoms. As this study was not a clinical trial but an exploratory preclinical investigation using genetically identical laboratory animals under standardized housing conditions, formal randomization procedures were not applied. All experimental interventions were performed by trained personnel with multi-year expertise. A.Ha. conducted all experiments following dedicated training, under the supervision of C.G. and A.He. for TI (> 10-year experience), L.W. and M.K. for splenectomy (> 15-year experience), and L.W., C.F., and M.M. for time-lapse MRI (> 10-year experience).

### Cell culture and TI

Murine breast cancer 4T1 and 67NR cells as established syngeneic murine breast cancer models [21, 22] were cultured in Dulbecco’s Modified Eagle Medium–(DMEM) + stable L-glutamine substitute–GlutaMAX (Thermo Fisher Scientific) with 10% fetal bovine serum (FBS) incubated under standardized conditions. Tumors were implanted by orthotopic injection of 1.0 × 10^6^ 67NR cells or 0.5 × 10^6^ 4T1 cells (cell numbers according to the different growth rates of 4T1 *versus* 67NR cells reflecting different grades of malignancy) resuspended in 50 µl cell culture medium into the lower mammary fat pad. Implantation of the tumor cells was performed under isoflurane inhalation anesthesia (1.5–2% isoflurane in 1 L O^2^/min). Tumor diameter was measured daily with a caliper.

### Immunotherapy and splenectomy

ICI therapy was started on day 5 after TI with intravenous injection of anti-mouse programmed death ligand 1 (PD-L1) (InVivoMAb, Bio X cell) every second day with a dose of 12.25 mg antibody per kg bodyweight. Splenectomy was performed according to Li et al [23]. Briefly, mice were anesthetized with isoflurane (induction with 5% in 1–2 l O^2^/min; maintenance with 1–2% in 1–2 L O^2^/min *via* a breathing mask) and given metamizole (200 mg/kg of body weight) for analgesia. After placement on a heat plate, mice were shaved, and the skin and the underlying fascial tissue were opened with a 0.5-cm incision. By carefully pulling the omentum, the spleen was placed outside the abdomen with its vessels carefully cauterized. Next, the omentum was placed back into the abdominal cavity, and both the fascial incision site, and the skin were closed with fine suture material. Before the mice were awakened under red light, tumor cells were injected. The analgesic treatment with metamizole was continued for 3 days (200 mg/kg of body weight subcutaneously). After surgery, aqua crystals and soft food (food pellets soaked in water) were given to the animals directly on the cage floor to facilitate food intake. Subsequently to performing splenectomy, tumor cells were injected as described above.

### MRI protocol and image analysis

*In vivo* time-lapse MRI of the murine brain was performed on a 9.4-T MRI (Biospec, Bruker Biospin) as recently described [19, 24]. Data was acquired using a cryogenic probe with a T2*-weighted gradient-echo sequence with following parameters: repetition time = 643.036 ms; echo time = 8.0 ms; flip angle 60°, number of excitations = 4; acquisition bandwidth = 50,000 Hz, acquisition matrix = 180 × 192; image matrix (reconstruction) = 180 × 256; in-plane resolution = 66 × 55 µm^2^; 38 contiguous slices; slice thickness = 300 µm, scan time = 8:12 min:s (single time frame), resulting in 2:44 h:min for 20 repetitions. Mice were anesthetized with 1.5% isoflurane in 1 L/min of oxygen and compressed air (1:4) under continuous respiratory and temperature monitoring. To avoid body cooling, mice were kept at physiologic temperature by a custom-designed animal heating device. A priori defined exclusion criteria for time-lapse MRI data analysis included pronounced physiological instability – such as significant changes in body temperature or respiratory rate—as well as the occurrence of unexpected motion artifacts. In such cases, measurements were stopped immediately and the corresponding datasets excluded from further analysis. For *in vivo* labeling of monocytes, Ferucarbotran (diameter 50–100 nm, Resovist, Bayer AG), a clinically approved standard iron oxide nanoparticles were injected intravenously in a volume of 3 mL/kg bodyweight (= 83.33 mg Fe per kg body weight) via the tail vein. Injection of the contrast agent was performed 24 h prior to the MRI scan to ensure clearing of free iron particles from the bloodstream [25]. Single iron-labeled cells detected by time-lapse MRI were identified as hypointense spots by two independent observers (A.Ha. and I.R.F.) to reduce observer-dependent bias, marked manually and tracked semiautomatically using ImageJ (Wayne Rasband, National Institute of Health) and a custom-made MATLAB script as described previously [24, 26–28]. The two readers (A.Ha. and I.R.F.) performed data analysis following structured training by M.M. (> 10-year experience in time-lapse MRI analysis). All readings were conducted blinded using pseudonymized animal identifiers; group allocation was only revealed after completion of the image evaluation. To assess interrater variability, a randomly selected data subset (*n* = 6) was independently analyzed by two readers. Corresponding Bland-Altman analysis is provided in supplementary Fig. S1.

### Tumor immune cell isolation and flow cytometric analysis

All mice were sacrificed after time-lapse MRI under deep anesthesia and tumors were harvested. To isolate immune cells, tumors were digested in 25-mM 4-(2-hydroxyethyl)-1-piperazineethanesulfonic acid–HEPES buffer (Carl Roth GmbH) containing collagenase (1 mg/mL collagenase A, Roche) and DNase I (50 μg/mL, Roche) for 45 min at 37 °C. The collagenase reaction was stopped by adding 10% FBS DMEM. Digested tissue was filtered through a 70-μm cell strainer (Merck, Bedford, USA); after centrifugation (350 g; 5 min; 4 °C), the cells were resuspended in fluorescence-activated cell sorting buffer. For the identification of TAM and pMO in tumor tissue, cells were stained with the following antibodies: CD45, CD11b, CD68, MHC II, CD206, PD-L1, Ly6C, CD8a (BioLegend) by incubation for 20 min at 4 °C. Thresholds were set with corresponding isotype controls. 4′,6-diamidino-2-phenylindole–DAPI (Thermo Fisher Scientific) staining was performed to exclude dead cells. After two washing steps, cells were resuspended in FACS buffer and flow cytometric analysis was performed on a CytoFLEX S (Beckman Coulter). Data were processed with FlowJo software (Version 10, BD Biosciences). The approach for identification of the immune cell subsets is provided in supplementary Fig. S2.

### Statistical analysis

Statistical analysis was performed using GraphPad Prism version 5 (GraphPad Software). Results shown are means ± standard error of the mean. Values were tested for normal distribution using the Shapiro–Wilk test. Unpaired *t*-test with Welch correction was performed for all MRI data, as well as flow cytometry data afterwards; *p*-values < 0.05 were considered statistically significant.

## Results

### Change in monocyte dynamics during tumor growth

**Fig. 3:**
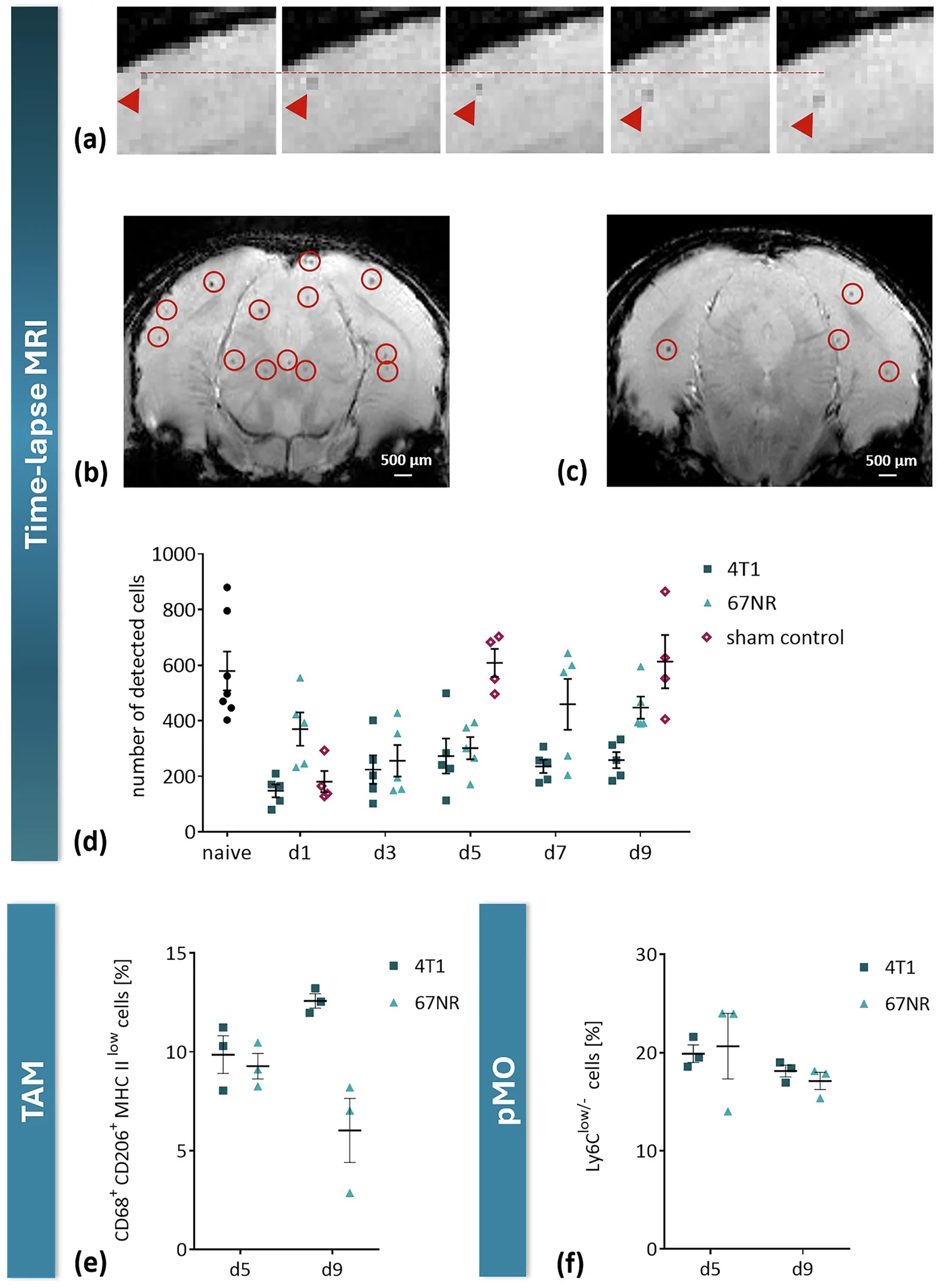
Changes in monocyte dynamics during tumor growth. **a** Representative image details showing consecutive time frames from one slice with an example of a single iron-labeled cell (red arrowheads) moving across several voxels (~ 0.2 µm/s). **b** Exemplary slice and time frame of a time-lapse MRI dataset of a brain from a naive control mouse showing multiple hypointense spots (highlighted by red circles) representing labeled monocytes. **c** Exemplary slice and time frame of a time-lapse MRI dataset of a brain from a 4T1 tumor-bearing mouse at day 7 post TI, showing fewer hypointense spots (highlighted by red circles). **d** Number of cells detected by time-lapse MRI of the brain at five time points post tumor initiation (*n* = 5) compared to naive control mice (*n* = 7) and sham control mice (*n* = 4). **e**, **f** *Ex vivo* flow cytometric analysis of the TME composition revealed no differences in TAM and pMO cell proportions (*n* = 3 each). Data are shown as mean ± standard error of the mean (detailed statistics in Supplementary Table S1). pMO, Patrolling monocytes; TAM, Tumor associated macrophages; TME, Tumor microenvironment

No unexpected adverse events occurred during the *in vivo* experiments, therefore, no animals or datasets were excluded from the analysis. The longitudinal time-lapse MRI in 4T1 or 67NR tumor-bearing mice revealed a change in monocyte dynamics during tumor growth. After TI, we observed a reduced number of detected cells (Fig. 3a, b). Quantitative analysis (Fig. 3c; detailed numbers and *p*-values in Table 1) revealed a significant reduction in cell counts in both 4T1- and 67NR tumor-bearing mice from day 1 to day 5 as compared to naive mice. Furthermore, a significant decrease in sham controls on day 1 compared to control mice was observed, attributable to an injection-related local inflammation known from previous studies [19]. By day 5 and day 9, however, the cell count in sham controls returned to levels observed in naive mice. Hence, time-lapse MRI is capable of depicting altered intravascular monocyte dynamics distant from the TME during early tumor growth. In contrast to 67NR tumor-bearing mice, 4T1 tumor-bearing mice showed a significantly lower number of detectable cells on day 7 and day 9 compared to naive mice. By day 9, the cell count in 4T1-bearing mice was significantly lower than in 67NR mice, indicating that the alteration in the number of monocytes detected by time-lapse MRI reflects the grade of malignancy. TME composition analyses by *ex vivo* flow cytometry showed that the proportion of TAMs did not differ significantly for 4T1 and 67NR tumors on day 5 or day 9 (Fig. 3d; detailed numbers and *p*-values in Table 2). The proportion of pMO cells within the TME was also similar for 4T1 and 67NR tumors on day 5 and day 9 (Fig. 3e, Table 2, and animal readouts are provided in Supplementary Table S1). Thus, the TME composition of the primary tumor did not correlate with the intravascular *in vivo* time-lapse MRI cell count in the brain.

**Table 1:** Number of detected cells with time-lapse MRI during tumor progression with corresponding statistics according to Fig. 3d

	Naive	Sham	4T1	67NR
d1	579 ± 69	181 ± 38	147 ± 23	370 ± 59
d3			224 ± 51	256 ± 56
d5		608 ± 50	273 ± 63	301 ± 40
d7			263 ± 23	459 ± 91
d9		612 ± 95	258 ± 29	447 ± 39

Unpaired *t*-test with Welch correction (*p*-values)				
d1	Naive	Sham	4T1	67NR
Naive	-	0.0009	0.0006	0.0466
Sham		-	0.4892	0.0348
4T1			-	0.0171
67NR				-

d3	Naive	Sham	4T1	67NR
Naive	-	-	0.0022	0.0050
Sham		-	-	-
4T1			-	0.6890
67NR				-

d5	Naive	Sham	4T1	67NR
Naive	-	0.7421	0.0089	0.0072
Sham		-	0.0044	0.0029
4T1			-	0.7160
67NR				-

d7	Naive	Sham	4T1	67NR
Naive	-	-	0.0021	0.3281
Sham		-	-	-
4T1			-	0.0694
67NR				-

d9	Naive	Sham	4T1	67NR
Naive	-	0.7869	0.0029	0.1359
Sham		-	0.0292	0.1863
4T1			-	0.0059
67NR				-

Data are shown as mean ± standard error of the mean; the Shapiro–Wilk test showed normal distribution, and *p*-values of unpaired t-test with Welch correction are given for statistical significances

**Table 2:** Flow cytometric analysis of tumor cell infiltrate with corresponding statistics according to Fig. 3e, f

	TAM [%]		pMO [%]	
	4T1	67NR	4T1	67NR
d5	9.8 ± 0.9	9.2 ± 0.6	19.8 ± 0.8	18.7 ± 1.9
d9	12.5 ± 0.3	6.0 ± 1.6	22.0 ± 2.1	16.4 ± 0.7

Unpaired *t*-test with Welch correction (*p*-values)				
TAM	4T1 d5	4T1 d9	67NR d5	67NR d9
4T1 d5	-	0.0884	0.6432	-
4T1 d9	-	-	-	0.0501
67NR d5	-	-	-	0.1726
67NR d9	-	-	-	-

pMO	4T1 d5	4T1 d9	67NR d5	67NR d9
4T1 d5	-	0.4194	0.6379	-
4T1 d9	-	-	-	0.1040
67NR d5	-	-	-	0.3663
67NR d9	-	-	-	-

Data are shown as mean ± standard error of the mean; the Shapiro–Wilk test showed normal distribution, and *p*-values of unpaired t-test with Welch correction are given for statistical significance

*pMO* Patrolling monocyte, *TAM* Tumor associated macrophage

### Change in monocyte dynamics after splenectomy

**Fig. 4:**
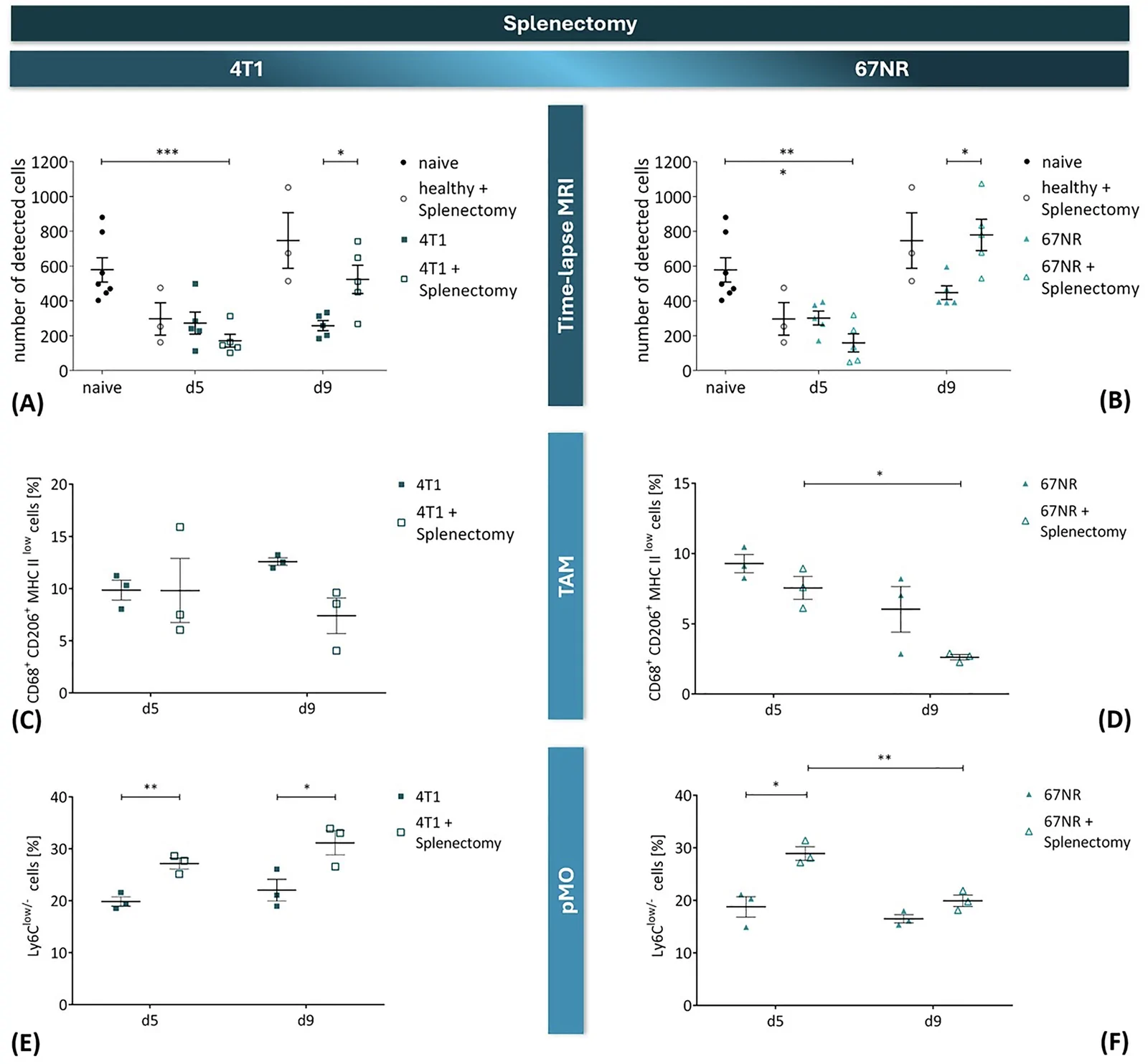
Changes in monocyte dynamics after splenectomy. **A**, **B** Number of detected cells by time-lapse MRI of the brain at day 5 or 9 after 4T1 (**A**) or 67NR (**B**) tumor initiation with or without splenectomy (*n* = 5 each), *versus* naive mice (*n* = 7) or mice receiving splenectomy without bearing breast cancer (*n* = 3, control). **C**–**F** *Ex vivo* flow cytometric analysis of the TME composition at day 5 and 9 regarding TAM (**C**, **D**) and pMO (**E**, **F**) cells in 4T1 (**C**, **E**) or 67NR (**D**, **F**) tumor-bearing mice with or without splenectomy (*n* = 3 each). Data shown as mean ± standard error of the mean (^*^p < 0.05, ^**^p < 0.01, ^***^p < 0.001). pMO, Patrolling monocytes; TAM, Tumor-associated macrophages; TME, Tumor microenvironment

To further explore the systemic regulation of intravascular monocyte dynamics, we next investigated the role of the splenic reservoir. The spleen is a major reservoir and source of circulating and tumor-infiltrating immune cells [29]. Thus, we aimed to investigate the importance of the spleen for the observed changes in intravascular monocyte dynamics during tumor growth. Monocyte dynamics and TME composition in either 4T1- or 67NR-bearing mice that received splenectomy immediately prior to TI were studied. Mice receiving splenectomy and no TI served as controls. In these control mice, the surgical procedure induced a slight, but non-significant decrease in the time-lapse MRI cell count on day 5 compared to naive mice, reflecting the influence of inflammatory triggers on intravascular monocyte dynamics (Fig. 4A, B; detailed numbers and *p*-values in Supplementary Table S2). On day 9, this was not observable anymore since monocyte counts recovered to an amount similar to naive mice. In tumor-bearing mice, splenectomy did not alter the number of detected cells on day 5 compared to mice without splenectomy. On day 9, however, splenectomy resulted in a significantly higher cell count in both 4T1 and 67NR mice compared to tumor-bearing mice without splenectomy. Thus, splenectomy reduced (4T1) or reversed (67NR) the effect of tumor growth-related alteration in intravascular monocyte dynamics in both low and highly malignant tumors. Again, we performed *ex vivo* TME composition analysis (detailed numbers including *p*-values provided in Supplementary Table S3, animal readouts are provided in Supplementary Table S1). Analysis did not show a significantly altered TAM population in mice with or without splenectomy in both 4T1 and 67NR tumors, respectively (Fig. 4C, D). However, the pMO population was significantly higher in mice with splenectomy in both 4T1 (day 5 and 9, Fig. 4E) and 67NR (day 5, Fig. 4F) tumor-bearing mice. Thus, the TME composition did not directly translate into the cell count observed by *in vivo* time-lapse MRI distant from the TME after splenectomy.

### Change in monocyte dynamics during immunotherapy

**Fig. 5:**
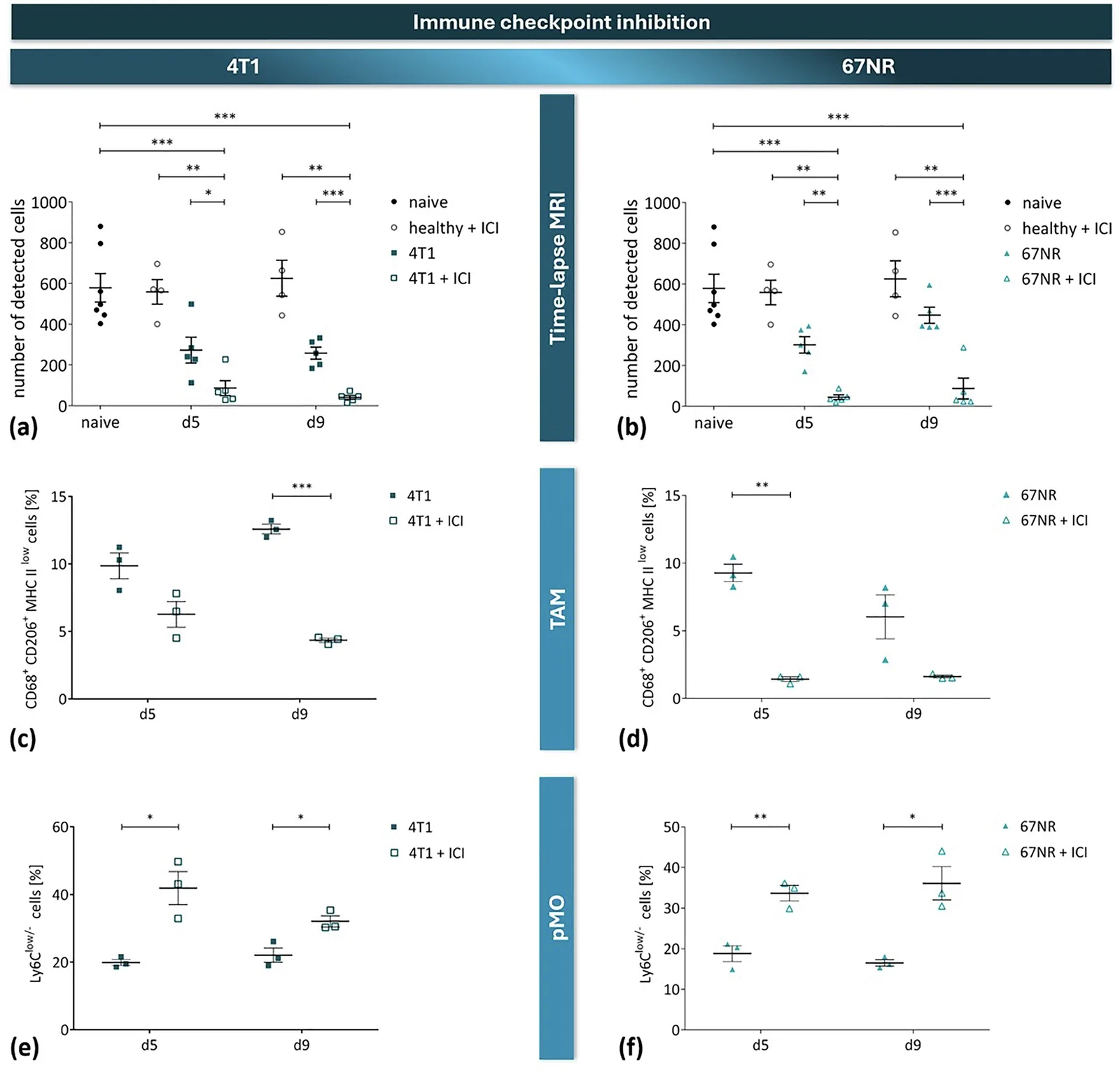
Changes in monocyte dynamics during immunotherapy. **a**, **b** Number of detected cells by time-lapse MRI of the brain at day 5 or 9 after 4T1 (**a**) or 67NR (**b**) TI with or without ICI (*n* = 5 each) *versus* naive mice (*n* = 7) or mice receiving ICI without bearing breast cancer (control, *n* = 4). **c**– **f** *Ex vivo* flow cytometric analysis of the TME composition on day 5 and 9 regarding TAM (**c**, **d**) and pMO (**e**, **f**) cells in 4T1 (**c**, **e**) or 67NR (**d**, **f**) tumor-bearing mice with or without ICI therapy (*n* = 3 each). Data shown as mean ± standard error of the mean (^*^p < 0.05, ^**^p < 0.01, ^***^p < 0.001). ICI, Immune checkpoint inhibitor; pMO, Patrolling monocytes; TAM, Tumor-associated macrophages; TME, Tumor microenvironment

Building on our observations during tumor growth and after splenectomy, we next sought to investigate how therapeutic modulation of the immune response influences intravascular monocyte dynamics. Given the clinical relevance of immunotherapy, we specifically examined whether ICI affects monocyte behavior at distant sites. 4T1- and 67NR tumor-bearing mice received ICI with anti-mouse PD-L1, whilst control mice without tumors received only ICI therapy. In both 4T1- and 67NR tumor-bearing mice, anti-PD-L1 therapy resulted in altered monocyte dynamics distant from the primary tumor site as revealed by time-lapse MRI (Fig. 5a, b; detailed numbers and *p*-values in Supplementary Table S4). In detail, cell counts in 4T1 and 67NR mice with ICI were significantly lower compared to tumor-bearing mice without ICI on day 5 and day 9 post-TI. Importantly, this effect was only observed if ICI was administered in tumor-bearing mice, while in control mice without tumors, ICI did not change the number of detected cells on day 5 or day 9 compared to naive mice. This shows that monocyte dynamics during ICI are only altered in the presence of a tumor and are not due to ICI per se, highlighting the potential of time-lapse MRI for therapy monitoring. Flow cytometry data (detailed numbers and *p*-values in Supplementary Table S5, animal readouts are provided in Supplementary Table S1) of the TME showed that ICI significantly reduced TAM abundance in both 4T1 (day 9) and 67NR (day 5) tumors (Fig. 5c, d). In contrast, the proportion of pMO cells within the TME significantly increased upon ICI both in 4T1 and 67NR tumors on day 5 and 9 post-TI (Fig. 5e, f). Again, the TME composition of the primary tumor did not directly translate into the cell count observed by *in vivo* time-lapse MRI distant from the TME during ICI.

## Discussion

We demonstrated in a murine breast cancer model that both cancer and immunotherapy alter slow intravascular “patrolling-like” monocyte motion at distant sites, with these changes linked to the tumor’s malignant potential and the presence of splenic reservoirs. We observed fewer monocytes exhibiting patrolling-like velocities in the brains of tumor-bearing mice. To achieve this, we used a clinically approved iron oxide nanoparticle as a contrast agent, that primarily labels monocytes [19] and time-lapse MRI to dynamically track single-cell motion [19, 20].

Compared to intravital microscopy, another dynamic single-cell imaging technique with excellent spatial resolution but an invasive character and a very small field-of-view, time-lapse MRI has been established as a noninvasive tool offering deep tissue imaging with a whole-organ view and excellent sensitivity [18–20, 24]. Due to limited temporal resolution, time-lapse MRI using Cartesian gradient-echo imaging depicts only cells with velocities up to that described for patrolling motion. Although several approaches increasing the detection limit have been presented [24, 30], the detection of rolling cells or even freely flowing cells within the bloodstream is currently not possible. Thus, time-lapse MRI is not intended to quantify the total number of monocytes in systemic circulation. Instead, it focuses on measuring the subset of cells exhibiting patrolling-like motion within a defined vascular bed. In this way, time-lapse MRI provides a motion-based imaging readout that reflects dynamic behavior consistent with patrolling, without necessarily identifying cells of a specific pMO phenotype. Nonetheless, our approach provides a new, dynamic, and real-time perspective at the single-cell level. It was shown before that immune cell tracking may serve as a potential biomarker of local or systemic inflammation, which is altered depending on the underlying stimulus and even before the onset of clinical symptoms [19]. In line with the recognized crosstalk between cancer and immunity, we provided evidence that single-cell tracking remote from the tumor site may contribute towards a more comprehensive view of cancer.

Our data confirm previous studies, since we also observed immediate changes in monocyte dynamics related to inflammatory stimuli such as cell or sham injection. However, cancer induced a continuing decrease in detectable monocytes within the brain, a vascular bed distant from the tumor site. This suggests that tumors of higher malignancy may drive a systemic shift in monocyte populations towards a protumoral subset, reducing their propensity for patrolling-like behavior at distant sites. The possible molecular mechanism of action involved in our observations should be subject to future studies.

Our data suggest that the splenic reservoir may contribute to TME-dependent alterations in intravascular monocyte dynamics during tumor growth. Following splenectomy, we observed a diminished impact on monocyte dynamics in the highly malignant cell line and a complete reversal in the low malignant cell line, compared to cancer growth in the presence of an intact spleen. Although we do not have direct insights into systemic monocyte availability, circulating subsets, or cytokine environments, our findings are consistent with a modulatory role of the spleen. The spleen, as the largest lymphoid organ, is a major source of myeloid-derived suppressor cells and has been the focus of several studies on cancer treatment using splenectomy. However, the impact of splenectomy on tumor growth remains inconclusive, with studies showing conflicting results: Some report that spleen removal promotes tumor growth [31, 32], others suggest an anti-tumor effect [33], while some find no significant impact [29]. Potentially, the impact of splenectomy on tumor growth depends on the timing of splenectomy relative to TI, on cancer cell type and stage [29, 31, 33, 34]. These studies found an alteration in circulating immune cells and TME composition. To ensure a consistent reduction of the splenic monocyte reservoir from the earliest phase of tumor development, we performed splenectomy immediately prior to TI. Our study is the first to focus analysis on the actual dynamic motion behavior of monocyte subsets post-splenectomy.

During immunotherapy, we observed the expected changes in TME composition, such as a decrease in the pro-tumoral TAM population and a significant increase in pMO as described previously [16], reflecting the intended therapeutic effect of the established immunotherapy and underlying the antitumoral role of pMO cells [15, 16]. In time-lapse MRI, we observed fewer cells with patrolling-like intravascular motion distant from the tumor under ICI. Importantly, this observation could not be attributed to a block of patrolling mechanisms by ICI *per se*, since ICI alone did not alter monocyte count in time-lapse MRI. This shows that the distant alteration in monocyte dynamics was dependent on anti-PDL1 interaction with the TME, emphasizing the early effect of the TME on systemic monocyte motion behavior during ICI and showing the potential of the technique for therapy monitoring. Importantly, while our *ex vivo* analysis of TME composition yielded consistent results reflecting the performed modulations by either splenectomy or ICI [29, 34–38], the TAM or pMO population did not correspond one-to-one with the time-lapse MRI cell counts in the brain. Rather, our data suggest that systemic intravascular monocyte patrolling-like motion is an independent variable influenced by multiple factors within the tumor-immune system interaction.

Our study has limitations. First, the relatively small group sizes and lack of correction for multiple comparisons, due to the exploratory nature of the study, warrant cautious interpretation of the results. Second, all experiments were conducted in a murine model of breast cancer using two well-established cell lines (4T1 and 67NR) that differ in their grade of malignancy, including their metastatic potential. However, our analysis does not account for the diversity of other tumors or subtypes of breast cancer, which may influence both tumor-immune interactions and systemic immune cell behavior. We thus acknowledge that the conclusions drawn here may not be generalizable to other tumor entities or molecular subtypes. Third, time-lapse MRI was performed only within the brain as a surrogate for a tumor-distant vascular bed and not within other organs or the TME itself. Applying time-lapse MRI to vascular beds more directly influenced by the tumor could help further elucidate the systemic immune response in cancer. The alteration of patrolling-like motion might differ within other vascular beds—regarding both cell velocity and effect size. In order to apply time-lapse MRI single-cell tracking to other organs or to the TME, major technical challenges to resolve motion and intrinsic tissue artifacts need to be addressed. While controlled *in vitro* systems like rotating phantoms [24, 30, 39] or new approach methodologies, including organ-on-chip systems, assembloids, and advanced *in vitro* co-culture models [40–43], may support such new fields of application in the future, currently time-lapse MRI studies require *in vivo* models to account for the highly complex and temporally evolving environment of cancer and cell motion. Fourth, our imaging readout is based on slow intravascular motion and serves as a surrogate for patrolling-like behavior, but does not allow for direct phenotypic identification of specific immune cell subsets. Moreover, changes in monocyte distribution, altered recruitment, or noncellular signaling processes involved in adhesion and patrolling may also contribute to the observed effects. Future studies should aim to clarify the specific immune cell phenotypes and mechanisms responsible for the detected patrolling-like motion. In this context, technical challenges must be overcome to advance time-lapse MRI capabilities for also tracking faster-moving cells. Although direct translation of time-lapse MRI to human application is currently limited by technical constraints, the approach may become feasible in the future, given the availability of clinically approved iron oxide-based contrast agents and high-field MRI systems (up to 11.7 T) for human use [44]. Additionally, accelerated image acquisition techniques hold promise for enabling these advancements in the future [24, 30].

In conclusion, using a preclinical time-lapse MRI-based single-cell tracking approach, we demonstrated in a syngeneic murine breast cancer model, that monocyte intravascular patrolling-like behavior is systemically altered at sites distant from the primary tumor, during tumor growth and immunotherapy, and that these changes depend on the tumor’s malignant potential and the existence of a splenic monocyte reservoir.

## Supplementary information

**Additional file 1: Supplementary Figure 1:** Interrater variability of detected cells by time-lapse MRI. Bland Altman Plot illustrating absolute differences and averages between the cell counts of first and second reader of randomly selected data subsets (n =6). The dashed red line represents the mean bias, the dashed black lines indicate 95% limits of agreement. **Supplementary Figure 2:** Flow cytometric analysis of the TME by immune cell isolation from murine breast tumors. Dot plots of gating strategy used for the identification of immune cell subpopulations in 4T1 and 67NR tumors. Cells were isolated from enzymatically digested tumors and after exclusion of cell debris and dead cells, leukocytes were identified by staining against the hematopoietic marker CD45. Macrophages were identified with CD45, CD11b and the panMØ marker CD68, tumor-associated macrophages (TAM) were characterized as M2 like CD206+ and MHC IIlow/- [36, 37]. Cytotoxic T-cells were identified based on the expression of CD8a+. Among CD11b+ cells, pMO were identified as Ly6Clow/-[13, 38]. Expression of PD-L1/CD274 within the TME was also analyzed. MØ – macrophages; pMO – patrolling monocytes; TAM – tumor associated macrophages; TME – tumor microenvironment. **Table S1.** Numbers of detected cells via time-lapse MRI of each animal. Given Data was used for creating graphs and calculating p-values. **Table S2.** Number of detected cells with time-lapse MRI during tumor progression and after splenectomy with corresponding statistics according to Figure 4(a) & 4(b). Data are shown in mean ± SEM, Shapiro-Wilk test showed normal distribution, p-values of unpaired t test with Welch´s correction are given for statistical significances. **Table S3.** Flow cytometric analysis of tumor cell infiltrate after splenectomy with corresponding statistics according to Figure 4(c), 4(d), 4(e), 4(f). Data are shown in mean ± SEM, Shapiro-Wilk test showed normal distribution, p-values of unpaired t test with Welch´s correction are given for statistical significances. **Table S4.** Number of detected cells with time-lapse MRI during tumor progression and ICI therapy with corresponding statistics according to Figure 5(a) & 5(b). Data are shown in mean ± SEM, Shapiro-Wilk test showed normal distribution, p-values of unpaired t test with Welch´s correction are given for statistical significances. ICI – immune checkpoint inhibitor. **Table S5.** Flow cytometric analysis of tumor cell infiltrate under ICI therapy with corresponding statistics according to Figure 5(c), 5(d), 5(e), 5(f). Data are shown in mean ± SEM, Shapiro-Wilk test showed normal distribution, p-values of unpaired t test with Welch´s correction are given for statistical significances. ICI – immune checkpoint inhibitor. **Supplementary Video S1:** Representative slice of a naive mouse brain acquired by time-lapse MRI with 20 time-frames 24 h after in vivo labelling of monocytes with iron oxide nanoparticles. Time-lapse MRI detects single cells with patrolling-like behavior displayed as hypointense spots. Detected cells are labelled with arrowheads. **Supplementary Video S2:** Representative slice of a naive mouse brain acquired by time-lapse MRI with 20 time-frames without injection of iron oxide nanoparticles showing negligible amounts of hypointensities.

## Data Availability

All data generated or analyzed during this study are included in this published article and its supplementary information files.

## Abbreviations

ICI: Immune checkpoint inhibitor
MRI: Magnetic resonance imaging
PD-L1: Programmed death-ligand 1
pMO: Patrolling monocyte
TAM: Tumor-associated macrophage
TI: Tumor implantation
TME: Tumor microenvironment
